# Overexpression of the MYB37 transcription factor enhances abscisic acid sensitivity, and improves both drought tolerance and seed productivity in *Arabidopsis thaliana*

**DOI:** 10.1007/s11103-015-0411-1

**Published:** 2015-12-08

**Authors:** Yong-Tao Yu, Zhen Wu, Kai Lu, Chao Bi, Shan Liang, Xiao-Fang Wang, Da-Peng Zhang

**Affiliations:** Center for Plant Biology, School of Life Sciences, Tsinghua University, Beijing, 100084 China

**Keywords:** Abscisic acid, *Arabidopsis thaliana*, Drought stress, Late flowering, MYB37 transcription factor, Seed production

## Abstract

Although a lot of genes have been revealed to participate in abscisic acid (ABA) signaling, many of the additional components involved in ABA signaling remain to be discovered. Here we report that overexpression of *MYB37*, a R2R3 MYB subgroup 14 transcription factor in *Arabidopsis thaliana*, confers hypersensitive phenotypes to exogenous ABA in all the major ABA responses, including ABA-induced inhibition of seed germination, cotyledon greening and early seedling growth, and ABA-induced stomatal closure and inhibition of stomatal opening. Interestingly and importantly, *MYB37*-overexpression improves plant tolerance to drought, enhances growth of mature plants and seed productivity, thought it delays flowering, which suggests that this gene may be used for improving crop adaptability to drought environment and productivity. However, a *myb37*-*1* knockout mutant displays wild-type ABA responses most likely due to a functional redundancy of the multiple MYB members. Real-time PCR analysis shows that upregulation of the *MYB37* expression changes expression of a subset of ABA-responsive genes. Together, these findings suggest that the MYB37 transcription factor plays an important, positive role in plant response to ABA and drought stress, and meanwhile, it plays a positive role in the regulation of seed production.

**Electronic supplementary material:**

The online version of this article (doi:10.1007/s11103-015-0411-1) contains supplementary material, which is available to authorized users.

## Introduction

Phytohormone abscisic acid (ABA) regulates many developmental processes including embryo maturation, seed germination and early seedling growth, and is a key hormone in plant adaptation to various adverse conditions such as drought, salt and cold stresses (Finkelstein et al. 2002; Himmelbach et al. 2003; Shinozaki et al. 2003; Adie et al. 2007; Cutler et al. 2010). Much progress has been made towards understanding the complicate ABA signaling network, and several types of ABA receptors, localized both at the cell surface and to intracellular space, have been identified (Shen et al. 2006; Ma et al. 2009; Park et al. 2009; Pandey et al. 2009; Santiago et al. 2009; Wu et al. 2009; Cutler et al. 2010; Shang et al. 2010; Du et al. 2012; Zhang et al. 2014). In addition to ABA receptors, numerous down-stream signaling components involved in various aspects of ABA response have been identified (Finkelstein et al. 2002; Himmelbach et al. 2003; Shinozaki et al. 2003; Adie et al. 2007; Cutler et al. 2010). These discoveries significantly deepen our understanding of ABA signaling from primary signal perception events to downstream gene expression. However, it is widely believed that ABA signal transduction involves highly complex signaling pathways, and additional components remain to be identified to fully understand the complex ABA signaling network.

Gene regulation through transcription factors (TFs) is the most important cell signaling process in plant response to ABA as well as changing environments (Zhu 2002; Shinozaki et al. 2003; Yamaguchi-Shinozaki and Shinozaki 2006). Several kinds of transcription factors, such as basic leucine zipper, WRKY, MYC, and NAC members, have been reported in abiotic stress response (Kang et al. 2002; Abe et al. 2003; Tran et al. 2004; Chen et al. 2012), and many members of the MYB transcription factor family have also been revealed to be involved in ABA signaling (Abe et al. 2003; Reyes and Chua 2007; Jung et al. 2008; Ding et al. 2009; Seo et al. 2009; Park et al. 2011; Zheng et al. 2012; Cui et al. 2013; Kim et al. 2015). MYB proteins contain a conserved DNA-binding MYB domain of about 52 amino acids. Based on the number of MYB domain, the MYB family can be divided into four classes, 1R-, R2R3-, 3R- and 4R-MYB proteins (Stracke et al. 2001; Dubos et al. 2010). The MYB family TFs comprise around 200 genes in *Arabidopsis thaliana* and are the largest TF gene family, representing about 9 % of the total TFs in *Arabidopsis*. About 126 MYB members belong to the R2R3-type subfamily. According to the conservation of the DNA binding domain and of amino acid motifs in the C terminal domains, R2R3-MYB proteins have been divided into 25 subgroups (Riechmann et al. 2000; Chen et al. 2006; Dubos et al. 2010). It has been shown that R2R3-MYB transcription factors are involved in various cell processes such as regulation of metabolism, modulation of development, determination of cell fate and response to biotic and abiotic stresses (Dubos et al. 2010; Pireyre and Burow 2015).

Although many MYB members have been reported to regulate ABA signaling and abiotic responses in *Arabidopsis*, there is no report, to our knowledge, that both stress tolerance and seed productivity may be improved at once by changes in expression of any *Arabidopsis* MYB member, which gives, however, particular significance in agriculture. Previous studies have shown that MYB37, a R2R3 MYB subgroup 14 transcription factor in *Arabidopsis,* regulates axillary meristem formation and vegetative development (Keller et al. 2006; Müller et al. 2006). Here we report that overexpression of *MYB37*, confers ABA-hypersensitivity in all the major ABA responses. *MYB37*-overexpression improves plant tolerance to drought, enhances growth of mature plants and productivity of seeds, thought it delays flowering, suggesting that this gene may be used for improving both crop adaptability to drought environment and productivity. These findings suggest that the MYB37 transcription factor plays an important, positive role in plant response to ABA and drought stress, and meanwhile, it plays a positive role in the regulation of seed production.

## Materials and methods

### Plant materials and growth conditions

*Arabidopsis* ecotype Col-0 was used as the wild-type control. *Arabidopsis MYB37* gene T-DNA insertion line (SALK_071748C) in the Col-0 ecotype background was obtained from the Arabidopsis Biological Resource Center (ABRC). The mutant line was genotyped by PCR, and the primer pairs LP-RP and RP-LBb1.3 were used for identifying the mutant. The sequences for these primers are presented in Supplemental Table 1. The mutant was designated *myb37*-*1*, and the mutant was previously identified as a null allele (Müller et al. 2006).

To generate transgenic plants overexpressing *MYB37* gene, the open reading frame (ORF) for the *MYB37* gene was isolated by PCR using primers presented in Supplemental Table 1. The ORF of *MYB37* was inserted into the pCAMBIA-1300-221 vector harboring green fluorescent protein (GFP)-encoding sequence (http://www.Cambia.Org/daisy/cambia/materials/vectors/585.html) at the *Xba*Ι and *Kpn*Ι sites under the control of a constitutive CaMV 35S promoter. The construct was verified by sequencing and introduced into the GV3101 strain of *Agrobacterium tumefaciens*. The construction was transformed by floral infiltration into Col-0 plants. Transgenic plants were selected by hygromycin resistance and confirmed by PCR. The homozygous T3 seeds of the transgenic plants expressing MYB37-GFP fusion protein were used for further analysis. We obtained 10 transgenic lines. All of the overexpression transgenic lines exhibited up-regulated *MYB37* expression profiles and hypersensitivity to ABA. We took *35S*-*MYB37* line 1 (OE1) and *35S*-*MYB37* line 6 (OE6) as representatives.

Plants were grown in a growth chamber at 21 °C on MS medium (Sigma) at ~80 μmol photons m^−2^ s^−1^or in compost soil at ~120 μmol photons m^−2^ s^−1^ cool-white fluorescent lamps under a 16 h-light/8 h-dark photoperiod and 60 % relative humidity.

### Real-time PCR analysis

Ten-day-old whole seedlings were used for determination of the *MYB37* transcript levels in the wild-type Col-0 and *MYB37*-overexpressing plants. Leaves of 3 to 4-week-old seedlings were sampled for ABA induction of *MYB37* and expression of a subset of ABA-responsive genes. Total RNA was isolated from 10-day-old plants or leaves of 3 to 4-week-old seedlings using Total RNA Rapid Extraction Kit (BioTeke, China), treated with RNase-free DNaseΙ (NEB) at 37 °C for 1 h to degrade genomic DNA, and purified by using RNA Purification Kit (BioTeke, China). Two micrograms of total RNA were subjected to first-strand cDNA synthesis using Roche Transcriptor First Strand cDNA Synthesis Kit and an oligo (dT18) primer. The primers of various ABA-responsive genes used for real-time PCR are listed in the Supplemental Table 2. Analysis was performed using the BioRad Real-Time System CFX96TM C1000 Thermal Cycler (Singapore). Amplification of ACTIN2/8 genes was used as an internal control. The cDNA was amplified using SYBR Premix Ex Taq (TaKaRa) with a DNA Engine Opticon 2 thermal cycler in a 10 μL volume. All experiments were repeated at least three times along with three biologically independent repetitions.

### Phenotypic analysis

For seed germination assay and cotyledon greening assay, about 100 seeds each from wild-type Col-0 plants and transgenic lines were disinfected and planted on MS medium. The medium contained 3 % sucrose and 0.8 % agar (pH 5.9) and supplemented with or without different concentrations of (±) ABA. The seeds were stratified at 4 °C for 3 days and then placed at 21 °C under light conditions. Germination was defined as an obvious emergence of the radical through the seed coat. Cotyledon greening was defined as obvious cotyledon expansion and turning green. Germination rates and cotyledon greening rates were recorded at the indicated times, respectively. Early seedling growth was assessed by directly planting the seeds in ABA-containing and ABA-free MS medium and investigations were done at indicated times.

To investigate ABA-induced stomatal closure, leaves of 3-week-old seedlings were floated in the buffer containing 50 mM KCl and 10 mM MES-KOH (pH 6.15) under a halogen cold-light source for 3 h followed by addition of different concentrations of (±) ABA. Stomatal apertures were scored on epidermal strips. To study ABA-inhibited stomatal opening, leaves of 3-week-old seedlings were floated on the same buffer and the plants were placed in the dark for 4 h before they were transferred to the cold-light for 2.5 h in the presence of different concentrations of (±) ABA and the stomatal apertures were recorded. For water-loss assays, rosette leaves of comparable size from 3 to 4-week-old plants grown under long days were detached, placed on a Petri dish and weighted at different times after detachment. For drought tolerance test, about 8-day-old seedlings were transplanted to the soil for 2 weeks under standard growth conditions, and then plants were subjected to progressive drought by withholding water for specified times and then re-watered for 2 days. The entire test was repeated five times.

### Subcellular localization of MYB37

Roots of 1-week-old *MYB37*-overexpressing transgenic seedlings (OE6) were immersed in 2 μg/mL 4′,6-diamidino-2-phenylindole (DAPI) solution for 10–15 min for nuclei labeling, and the seedlings were observed by confocal microscope (Zeiss LSM780, Germany).

## Results

**Fig. 1:**
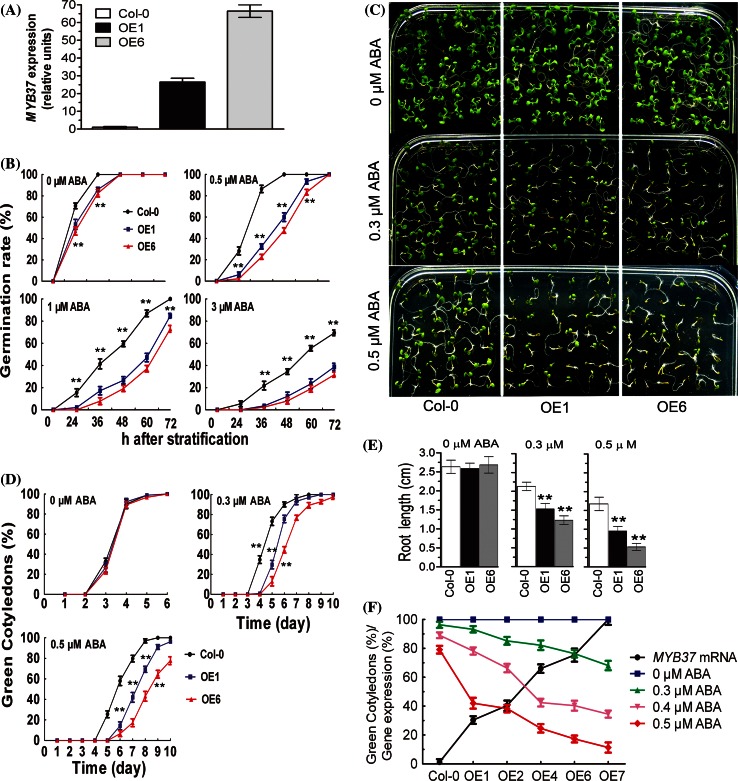
Germination and early seedling growth of the *MYB37* overexpression lines in response to ABA. **a** Determination of the *MYB37* transcript levels in the wild-type Col-0 plants and two *MYB37* overexpression lines (OE1 and OE6) by real-time PCR. “Relative units” for the *MYB37* expression are normalized relative to the value of wild-type Col-0, which is taken as 1. Each value is the mean ± SE of three independent biological determinations. **b** Seed germination. Germination rates of the wild-type Col-0 and two *MYB37*-overexpressing lines (OE1 and OE6) were scored on ABA-free (0 μM) and ABA-containing (0.5, 1 or 3 μM) medium from 24 to 72 h after stratification. Each value is the mean ± SE of three biological determinations. Student’s *t* test was used to compare the germination rates of each transgenic line with those of the wild type Col-0 (with significant differences at **P < 0.01). **c** Early seedling growth. Seeds of the wild-type Col-0, OE1 and OE6 plants were directly planted in MS medium supplemented with different concentrations of (±)ABA (0, 0.3, or 0.5 μM), and the growth was investigated 9 days after stratification. **d** Statistics of the rates of green cotyledons of the plants as described in (**c**). Green cotyledons were recorded at the indicated times. Each value is the mean ± SE of 100 seeds from three independent experiments. Student’s *t* test was used to compare the percentages of green cotyledons of each transgenic line with those of the wild type Col-0 (with significant differences at **P < 0.01). **e** Statistics of the primary root lengths of the plants as described in (**c**). Student’s *t* test was used to compare the primary root lengths of each transgenic line with those of the wild type Col-0 (with significant differences at **P < 0.01). (**f**) Percentages of green cotyledons of the wild-type Col-0 plants and different *MYB37* overexpression lines in the MS medium supplemented with different concentrations of (±)ABA (0, 0.3, 0.4 and 0.5 μM). Green cotyledons were scored 7 days after stratification. The *MYB37* mRNA levels in the Col-0 plants and *MYB37* overexpression lines (OE1, OE2, OE4, OE6 and OE7) are shown along with their data of the percentages of green cotyledons to display clearly the negative correlation of the *MYB37* mRNA levels with the percentages of green cotyledons in the ABA-containing medium. The *MYB37* mRNA levels in the Col-0 plants and *MYB37* overexpression lines are relative units, normalized relative to the mRNA level of the OE7 with the highest *MYB37* mRNA (taken as 100 %). Values are the mean ± SE from three independent experiments

Overexpression of *MYB37* enhances ABA sensitivity in the major ABA responses and improves plant tolerance to drought. Seeds of the *MYB37*-overexpressing transgenic lines (OE1 and OE6, Fig. 1a) germinated slowly than the wild-type seeds in the ABA-free medium (Fig. 1b), suggesting that transgenic seeds are likely to be more sensitive to endogenous ABA that the wild-type seeds. In the presence of different concentrations of exogenous ABA (0.5, 1 or 3 μM), the germination of OE1 and OE6 seeds was considerably delayed, showing significantly ABA hypersensitive phenotype compared with the wild-type seeds (Fig. 1b). Also, early growth of the OE1 and OE6 seedlings was significantly reduced than that of the wild-type seedlings in the ABA-containing medium (Fig. 1c). Cotyledon greening is one of the key developmental events during post-germinative growth and enables a seedling to establish photosynthetic capacity. In the absence of ABA, the cotyledon-greening percentages and primary root length of the wild-type, OE1 and OE6 plants were similar (Fig. 1d). In the presence of different concentrations of ABA (0.3 and 0.5 μM), OE1 and OE6 plants showed significantly decreased cotyledon-greening percentages and primary root length than wild-type plants, showing ABA hypersensitive phenotypes compared with the wild-type plants (Fig. 1d, e). Using a series of the *MYB37*-overexpressing transgenic lines, we showed that the amounts of *MYB37*-expression levels are negatively correlated with the cotyledon-greening rates (Fig. 1f), supporting that MYB37 positively regulates ABA signaling.

**Fig. 2:**
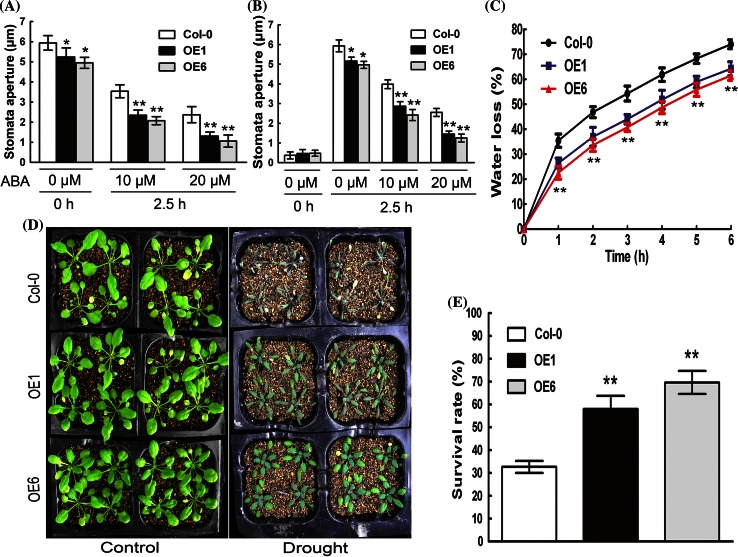
Stomatal movement and drought tolerance of the *MYB37* overexpression lines in response to ABA. **a** ABA-induced stomatal closure, and **b** ABA-induced inhibition of stomatal opening for the wild-type Col-0, OE1 and OE6 plants. Mature rosette leaves from 3-week-old seedlings were used for the assays. Values are the mean ± SE from three independent experiments; 60 apertures were counted for every experiment. Values are the mean ± SE from three independent experiments (n ≥ 60 apertures per experiment), and Student’s *t* test was used to compare means between Col-0 and transgenic plants within the same ABA concentration (with significant differences at *P < 0.05 or **P < 0.01). **c** Water loss rates during a 6-h period from the detached leaves of the wild-type Col-0, OE1 and OE6 plants. Each value is the mean ± SE of three biological determinations. Student’s *t* test was used to compare the rates of water loss of each transgenic line with those of the wild type Col-0 (with significant differences at **P < 0.01). **d** Whole-plant status in a drought tolerance assay for the wild-type Col-0, OE1 and OE6 plants. Plants were well watered (Control) or drought stressed by withholding water (Drought) for 2 weeks. The experiments were repeated five times with similar results. **e** The survival rates of the wild-type Col-0, OE1 and OE6 plants after drought for 2 weeks and re-watering for 2 days. Student’s *t* test was used to compare the survival rates of each transgenic line with those of the wild type (**P < 0.01)

The promotion of stomatal closure and inhibition of stomatal opening have been believed to be two distinct ABA-mediated processes, which can minimize water transpiration from the leaves under drought conditions (Zhu 2002; Kwak et al. 2008). Stomatal apertures were investigated in 10 and 20 μM (±) ABA treated leaves of the wild-type, OE1 and OE6 plants (Fig. 2a, b). Stomata of OE1 and OE6 transgenic plants had smaller apertures than did wild-type plants (Fig. 2a, b), suggesting that transgenic stomata is likely to be more sensitive to endogenous ABA that the wild-type stomata, which is consistent with the above-mentioned observations in seed germination (Fig. 1b). In the presence of exogenously applied ABA, we showed that the OE1 and OE6 plants exhibited significantly ABA-hypersensitive phenotypes in both ABA-induced promotion of stomatal closure and inhibition of stomatal opening compared with wild-type plants (Fig. 2a, b). Consistently, under dehydration conditions, the detached leaves of OE1 and OE6 lost less water than those of wild-type plants (Fig. 2c), which suggests that OE1 and OE6 had higher capacity to conserve their water compared with wild-type plants. So we further assayed whether OE1 and OE6 transgenic plants were drought-tolerant. Three-week-old plants were subjected to water deficit conditions by withholding water for 2 weeks. Under the well-watered condition, wild-type, OE1 and OE6 plants showed comparable growth vigor (Fig. 2d), but the wild-type plants showed severe wilting symptoms after 2 weeks of drought treatment compared with OE1 and OE 6 plants (Fig. 2d). The drought-treated plants were re-watered for 2 days and their survival rates were determined, which showed that the OE1 and OE6 plants exhibited higher survival rates, more drought-tolerant than wild-type plants (Fig. 2e).

We showed that the GFP-expressing plants displayed wild-type ABA responses (Supplementary Fig. S1), indicating that the observed ABA-related phenotypes of the transgenic plants expressing the MYB37-GFP fusion protein are specific and reliable. In addition, the endogenous ABA concentrations and expression levels of a subset of ABA metabolism-related genes of the *MYB37*-overexpressing transgenic lines were shown to be comparable to those of wild-type plants (Supplementary Fig. S2), indicating that the observed ABA-related phenotypes are independent of endogenous ABA concentrations.

### Overexpression of *MYB37* promotes growth of mature plants and productivity of siliques, thought it delays flowering

**Fig. 3:**
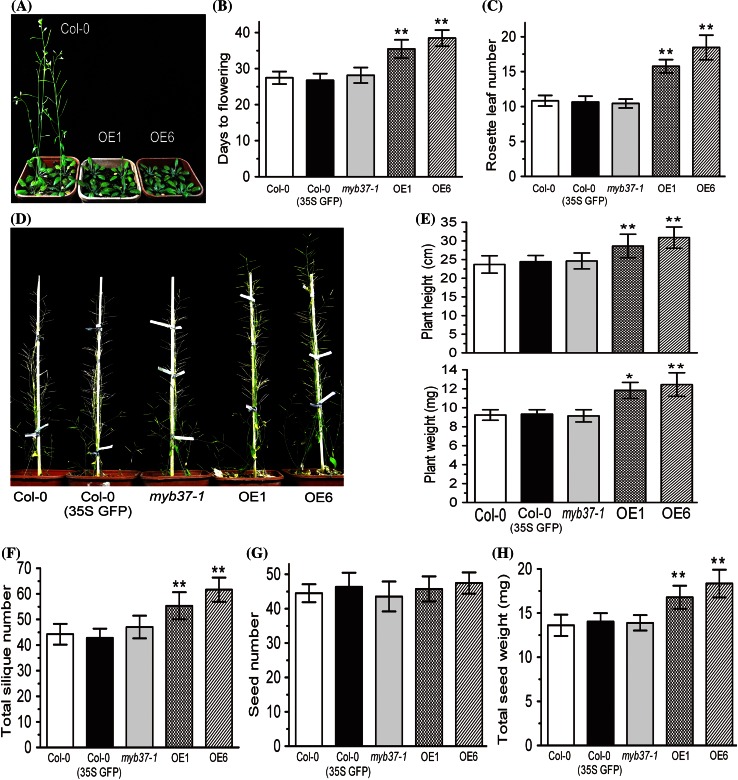
*MYB37* overexpression delays flowering, but improves growth of mature plants and productivity of siliques. **a** The wild-type Col-0, OE1 and OE6 plants grown for 5 weeks under long days (16 h light/8 h dark). **b** Statistics of the time (days) required for flowering of the wild-type Col-0, transgenic plants expressing GFP [Col-0 (35S GFP)], *myb37*-*1*, OE1 and OE6 plants. Student’s *t* test was used to compare the flowering time of different genotypes with that of the wild type (**P < 0.01). **c** Statistics of the rosette leaf numbers at the flowering stage of the wild-type Col-0, Col-0 (35S GFP), *myb37*-*1*, OE1 and OE6 plants. Student’s *t* test was used to compare the rosette leaf numbers of different genotypes with that of the wild type (**P < 0.01). **d** The mature plants of wild-type Col-0, transgenic lines expressing GFP [Col-0 (35S GFP)], *myb37*-*1* mutant, OE1 and OE6 lines (grow for 11  weeks under the 16 h light/8 h dark condition). **e** Statistics of the major stem height (plant height, top) and the main stem weight (dry weight) per plant (plant weight, bottom) of the wild-type Col-0, Col-0 (35S GFP), *myb37*-*1*, OE1 and OE6 plants. Student’s *t* test was used to compare the major stem height and main stem weight of different genotypes with that of the wild type (*P < 0.05; **P < 0.01). **f** Statistics of the total silique members per plant of the wild-type Col-0, Col-0 (35S GFP), *myb37*-*1*, OE1 and OE6 plants. Student’s *t* test was used to compare the total silique members per plant of each genotype with those of the wild type (**P < 0.01). **g** Statistics of the seed members per silique of the wild-type Col-0, Col-0 (35S GFP), *myb37*-*1*, OE1 and OE6 plants. Student’s *t* test was used to compare the seed members per silique of each genotype with those of the wild type, but no significant difference was found. **h** Statistics of the total seed weight (dry weight) per plant of the wild-type Col-0, Col-0 (35S GFP), *myb37*-*1*, OE1 and OE6 plants. Student’s *t* test was used to compare the total seed weight per plant of each genotype with that of the wild type (**P < 0.01)

Under long day conditions the appearance of flower buds in the OE1 and OE6 plants was delayed 8 and 12 days, respectively, compared with wild-type plants, as evidenced by measurements of time (days) required for flowering and rosette leaf number when flowering (Fig. 3a–c). However, the mature transgenic OE1 and OE6 plants catch up with and exceed wild-type plants in their growth during the late developmental stages, displayed significantly higher stem height and weight or bigger biomass than wild-type plants (Fig. 3d, e). Interestingly and importantly, the transgenic OE1 and OE6 plants produced more siliques (Fig. 3f) with the same size (Fig. 3g) as comparison with the wild-type plants, which resulted in higher production of seeds of the transgenic plants, as estimated by total weight of seeds per plant (Fig. 3h).

### Disruption of *MYB37* affects neither ABA response nor plant growth/production

**Fig. 4:**
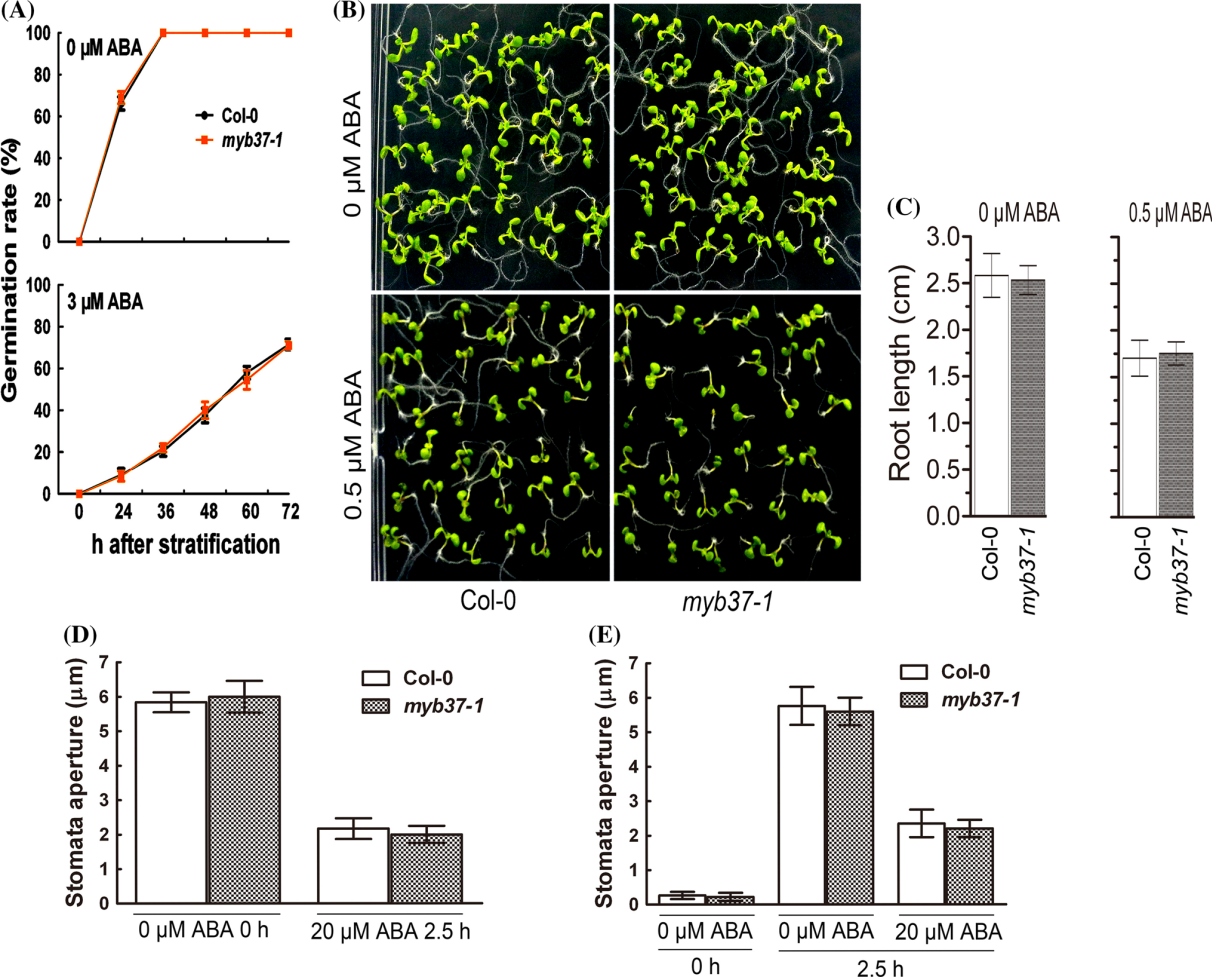
Knockout mutant of the *MYB37* gene shows wild-type response to ABA. **a** Seed germination rates of the *myb37*-*1* mutant. Germination rates of the wild-type Col-0 and *myb37*-*1* were scored on ABA-free (0 μM) and ABA-containing (3 μM) medium from 24 to 72 h after stratification. Each value is the mean ± SE of three biological determinations. No significant difference was found between the wild-type Col-0 and *myb37*-*1* plants at the level of *P* < 0.05 (Student’s *t* test). **b** Early seedling growth of *myb37*-*1* mutant. Seeds of the wild-type Col-0 and *myb37*-*1* plants were directly planted in ABA-free medium and a medium containing 0.5  μM (±)ABA, and the growth was investigated 9 days after stratification. **c** The primary root lengths of the plants as described in (**b**). No significant difference was found between the wild-type Col-0 and *myb37*-*1* plants at the level of *P* < 0.05 (Student’s *t* test). **d** ABA-induced stomatal closure and **e** inhibition of stomatal opening for the wild-type Col-0 and *myb37*-*1* plants. Values are the mean ± SE from three independent experiments; 60 apertures were counted for every experiment. No significant difference was found between the wild-type Col-0 and *myb37*-*1* plants at the level of *P* < 0.05 (Student’s *t* test)

We tested the ABA-related phenotypes of a null mutant *myb37*-*1*, but we found that the *myb37*-*1* mutant did not exhibit any ABA-related phenotype during seed germination, early seedling growth and ABA-induced promotion of stomatal closure and inhibition of stomatal opening (Fig. 4a–e). In addition, the *myb37*-*1* mutant affected neither plant growth nor seed production (Fig. 3b–h). This is most likely due to a functional redundancy of the multiple MYB members.

### Expression profile of the *MYB37* gene and subcellular localization of the MYB37 protein

**Fig. 5:**
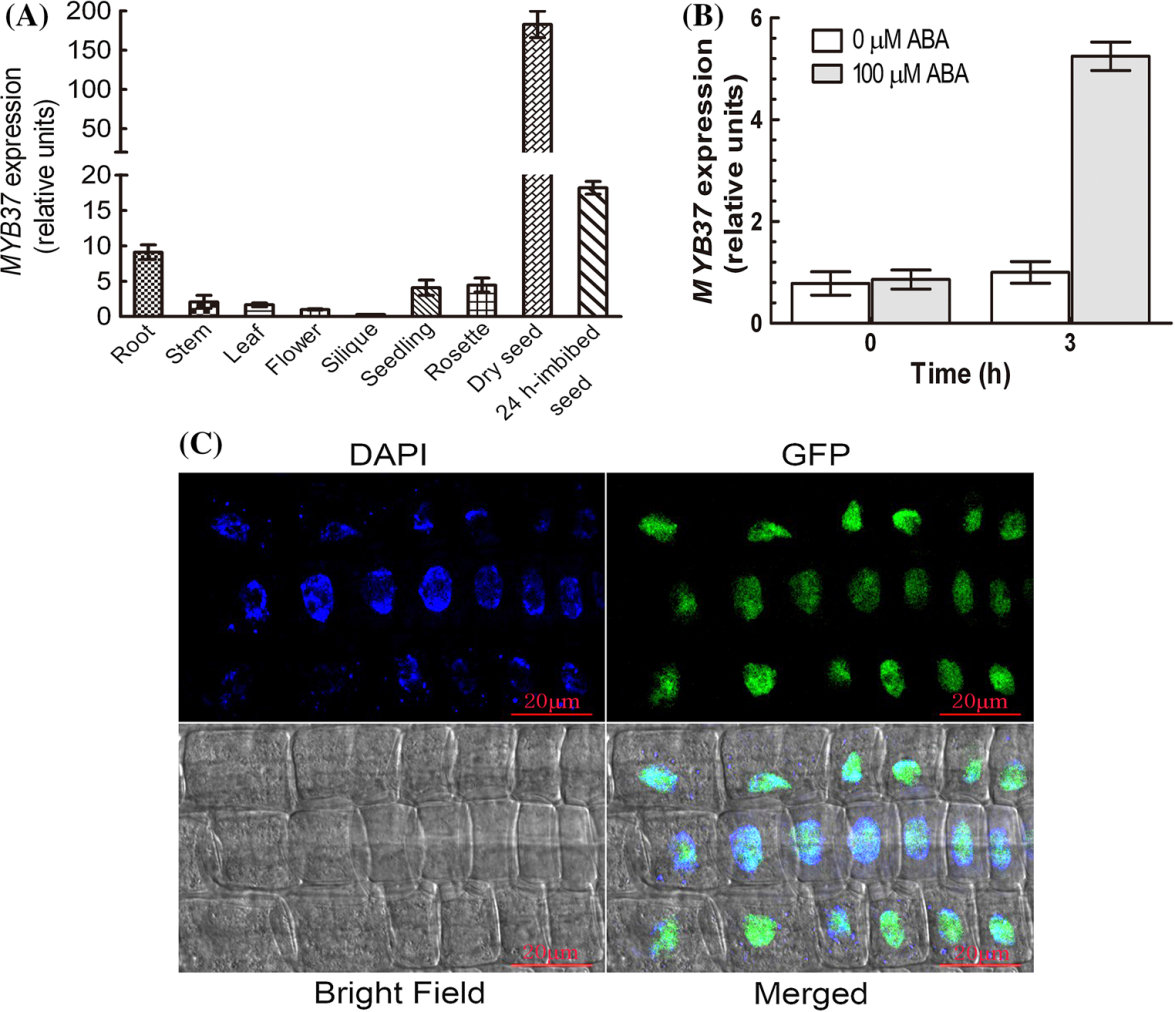
Expression profiles of *MYB37*. **a** Expression of *MYB37* in various tissues, determined by real-time PCR. “Relative units” for the *MYB37* expression are normalized relative to the value of flower, which is taken as 1. **b** ABA treatment enhanced the expression levels of *MYB37*, which were determined by real-time PCR. The wild-type Col-0 seedlings grown in soil (3–4-week-old) were sprayed with 100 μM (±)ABA solution or mock solution [0 μM (±)ABA, as a control], and sampled for analysis 3 h after the spraying. “Relative units” for the *MYB37* expression are normalized relative to the value of the 0 μM (±)ABA treatment for 3 h, which is taken as 1. **c** Confocal microscopy images of the GFP-tagged MYB37 in the root cells of the transgenic line OE6. A portion of the MYB37-GFP fusion protein (GFP, *top-right panel*) and a nuclear marker DAPI (*top-left panel*) are co-localized to the nucleus (Merged, *bottom-right panel*) with the bright field shown in the *bottom-left panel*. The experiments were repeated three times with the same results. *Bars* 20 μm

*MYB37* was shown to be expressed in different organs/tissues but more abundantly in dry seed, 24 h-imbibed seed and root than other tissues (Fig. 5a). This is consistent with previous report that *MYB37* is more abundantly expressed in root than leaf and flower (Müller et al. 2006). Furthermore we observed that the expression of *MYB37* in seedlings was induced by ABA (Fig. 5b).

We used a *MYB37*-overexpressing line OE6 to examine the subcellular localization of MYB37, which expresses the MYB37-GFP fusion protein. The roots (elongation zone) of *Arabidopsis* transgenic plants showed high GFP activities in the nuclear regions (Fig. 5c), revealing that MYB37 is a nuclear-localized protein, consistent with a transcription factor.

### Overexpression of *MYB37* alters the expressions of a subset of ABA-responsive genes

**Fig. 6:**
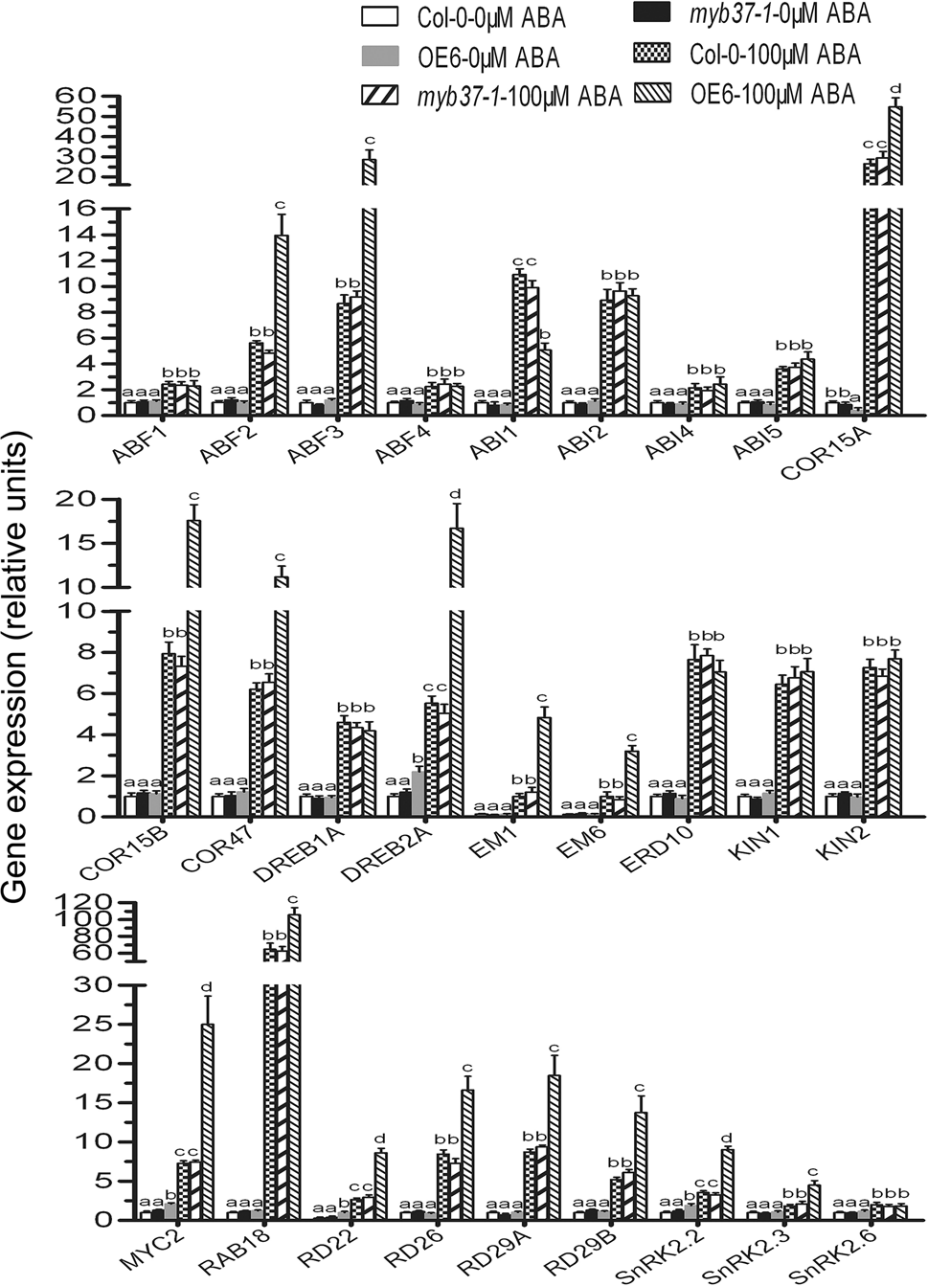
Expression of a subset of ABA-responsive genes is altered in *MYB37*-overexpressing plants. Gene expression in the 4-week-old wild-type Col-0, *myb37*-*1* and OE6 plants was determined by real-time PCR. Plants were sprayed with 100 μM (±)ABA solution or mock solution [0 μM (±)ABA solution, as a control], and sampled for gene expression analysis 3 h after the ABA treatment. “Relative units” for the gene expression are normalized relative to the value of wild-type with 0 μM (±)ABA treatment, which is taken as 1. Each value is the mean ± SE of three biological determinations, and *different letters* indicate significant differences at *P* < 0.05 (Duncan’s multiple range test) when comparing the expression levels for the same gene in Col-0, *myb37*-*1* and OE6 plants

Real-time PCR experiments were conducted to compare the transcript levels of a subset of ABA-responsive genes in the wild-type, *myb37*-*1* and OE6 plants using gene specific oligonucleotide primers presented in Supplemental Table 2. These assayed ABA responsive genes were: *ABFs* (*ABF1*, *ABF2*/*AREB1*, *ABF3* and *ABF4*/*AREB2*) (Choi et al. 2000; Uno et al. 2000; Kang et al. 2002; Yoshida et al. 2010), *ABI1* (Leung et al. 1994; Meyer et al. 1994), *ABI2* (Leung et al. 1997), *ABI4* (Finkelstein et al. 1998), *ABI5* (Finkelstein and Lynch 2000), *COR15A*, *COR15B* and *COR47* (Gilmour et al. 1998; Sakuma et al. 2006), *DREB1A* and *DREB2A* (Liu et al. 1998; Sakuma et al. 2006), *EM1* and *EM6* (Gaubier et al. 1993; Devic et al. 1996), *ERD10* (Kiyosue et al. 1994), *KIN1* and *KIN2* (Kurkela and Borg-Franck 1992), *MYC2* (Abe et al. 2003), *RAB18* (Lang and Palva 1992), *RD22* (Yamaguchi-Shinozaki and Shinozaki 1993), *RD26* (Fujita et al. 2004), *RD29A* and *RD29B* (Yamaguchi-Shinozaki and Shinozaki 1994), and three *SnRKs* (*SnRK2.2*, *SnRK2.3*, *SnRK2.6*/*OST1*) (Fujii and Zhu 2009). The expressions of sixteen positive ABA signalling regulator-encoding (or positively ABA-responsive genes) genes (*ABF2*, *ABF3*, *COR15A*, *COR15B*, *COR47*, *DREB2A*, *EM1*, *EM6*, *MYC2*, *RAB18*, *RD22*, *RD26*, *RD29A*, *RD29B*, *SnRK2.2* and *SnRK2.3*) were significantly up-regulated in the ABA-treated OE6 plants compared with wild-type plants (Fig. 6), while the expression of one negative ABA signaling regulator-encoding gene *ABI1* was repressed in the ABA-treated OE6 plants (Fig. 6). Additionally, the transcript levels of *DREB2A*, *MYC2*, *RD22* and *SnRK2.2* were also significantly up-regulated in the OE6 plants in the absence of the exogenous ABA treatment (Fig. 6). These findings are essentially consistent with the positive role of MYB37 in ABA signaling. However, the expression levels of all the assayed ABA-responsive genes of  the *myb37-1* mutant were comparable to those of wild-type in either the absence or presence of the exogenous ABA treatment (Fig. 6), supporting the functional redundancy of the multiple MYB members. The expression levels of the ABA-responsive genes *ABF1*, *ABF4*, *ABI2*, *ABI4*, *ABI5*, *DREB1A*, *ERD10*, *KIN1*, *KIN2* and *SnRK2.6* were not affected by *MYB37*-overexpression (Fig. 6), suggesting that these genes may not be involved in the *MYB37*-medaited ABA signaling.

## Discussion

MYB proteins are key regulators involved in plant response to biotic and abiotic stresses (Dubos et al. 2010; Pireyre and Burow 2015). A lot of MYB transcription factors are reported to be involved in ABA or/and abiotic stress responses in *Arabidopsis*. Five MYB members, MYB2, MYB7, MYB30, MYB33 and MYB101, are involved in ABA signaling during germination or/and seedling growth (Abe et al. 2003; Reyes and Chua 2007; Zheng et al. 2012; Kim et al. 2015). Five other MYB members, MYB15, MYB20, MYB44, MYB52 and MYB96 are involved in ABA and drought, salt or/and cold responses (Agarwal et al. 2006; Huang et al. 2007; Jung et al. 2008; Ding et al. 2009; Seo et al. 2009, 2011 Park et al. 2011; Cui et al. 2013; Jaradat et al. 2013; Persak and Pitzschke 2013; Gao et al. 2014; Lee et al. 2015; Lee and Seo 2015). Additionally, twelve MYB members, including MYB21, MYB41, MYB60, MYB61, MYB73, MYB75, MYB88, MYB102, MYB108 (BOS1), MYB124, MYBC1 (a single-repeat R3-MYB member) and HOS10 (a R2R3-type MYB member), participate in drought, salt or/and cold responses (Denekamp and Smeekens 2003; Mengiste et al. 2003; Cominelli et al. 2005, 2008; Zhu et al. 2005; Zhai et al. 2010; Xie et al. 2010; Oh et al. 2011; Romano et al. 2012; Kim et al. 2013; Su et al. 2013; Nakabayashi et al. 2014).

Among these MYB members involved in ABA or/and abiotic stress responses, overexpression of MYB15, MYB44, MYB52, MYB61, MYB75 and MYB96, respectively, has been reported to improve drought or/and salt tolerance (Jung et al. 2008; Ding et al. 2009; Seo et al. 2009, 2011; Park et al. 2011; Romano et al. 2012; Nakabayashi et al. 2014), while loss-of-function of MYB60 or MYB73 also resulted in drought or/and salt tolerance (Cominelli et al. 2005; Kim et al. 2013). In most cases, however, tolerance to abiotic stresses of the transgenic overexpressors or loss-of-function mutants may be combined to reduction of their growth or/and loss of seed productivity. For instance, overexpression of MYB52 or MYB96 conferred dwarf phenotypes, and overexpression of MYB44 or MYB61 decreased seed productivity, thought overexpression of these genes improved tolerance to drought or salt stress (Jung et al. 2008; Seo et al. 2009; Park et al. 2011; Romano et al. 2012). There is no report, to our knowledge, that over- or down-regulation of any *Arabidopsis* MYB member improves both tolerance to abiotic stress and seed productivity.

In the present study, we showed that the *MYB37*-overexpressors exhibited ABA hypersensitivity in all the major ABA responses in *Arabidopsis*, including ABA-induced inhibition of seed germination, cotyledon greening and early seedling growth, and ABA-induced promotion of stomatal closure and inhibition of stomatal opening (Figs. 1, 2). Consistently with the hypersensitivity of guard cells in response to ABA, the *MYB37*-overexpressing transgenic lines displayed significantly reduced water loss rate and enhanced drought tolerance (Fig. 2). These findings suggest that MYB37 positively regulates ABA signaling and plant response to drought. Changes of the expression of a subset of ABA responsive genes in the *MYB37*-overexpressors (Fig. 6) support this suggestion. Most interestingly and importantly, the *MYB37*-overexpressors showed increased biomass production and improved seed productivity, as estimated, respectively, by total height and weight of mature plants and silique number/seed total weight per plant (Fig. 3), which suggest that MYB37 is likely to be useful in crop improvement by transgenic manipulation to improve crop tolerance to drought environment while promoting crop production.

In regard to the mechanism to explain the improved drought tolerance in the *MYB37*-overexpressing lines, the enhanced sensitivity of stomatal closure to ABA by *MYB37* overexpression (Fig. 2) can minimize water transpiration from leaves under water-deficit conditions that induce ABA accumulation (Leung and Giraudat 1998; Zhu 2002; Kwak et al. 2008). However, ABA regulates plant adaptation to water deficit through regulating both water balance and osmotic stress/cellular dehydration tolerance. Whereas the function of ABA in water balance is mainly through guard cell regulation, the latter role is related to the induction of genes that encode dehydration tolerance proteins in nearly all cells (Zhu 2002). MYB37-overexpression leads to upregulation of expression of a number of ABA-responsive, stress tolerance–related genes such as *ABF2*, *ABF3*, *DREB2A* and *MYC2* (Fig. 6), suggesting that MYB37 may also function at the level of cellular dehydration tolerance.

It is noteworthy that the null mutant *myb37*-*1* showed wild-type response to ABA (Fig. 4). This is most likely because of the functional redundancy among the multiple MYB members. The group of the R2R3-MYB proteins, to which MYB37 belongs, includes 25 subgroups in *Arabidopsis*. MYB37 is a member of subgroup 14, which is most homologous to the subgroup 1. So the functional redundancy may be produced among members of subgroup 14 or between subgroup 14 and subgroup 1. It has been known that the MYB proteins of the subgroup 1 play relatively the most important roles in ABA signaling pathways among 25 subgroups (Seo et al. 2009; Zheng et al. 2012; Lee et al. 2015). MYB96, a member of subgroup1, functions similarly to MYB37, as a positive regulator of ABA signaling in seed germination and stomatal movement, and plays a positive role in plant response to drought stress (Seo et al. 2009; Lee et al. 2015). Whereas the *myb37*-*1* single mutant has wild-type ABA responses as we observed in the present study (Fig. 4), the single loss-of-function mutants of some *MYB* genes of the subgroup 1, such as *MYB30* and *MYB96*, exhibit altered ABA-related phenotypes (Seo et al. 2009; Zheng et al. 2012; Lee et al. 2015). Some R2R3-MYB members belonging to the subgroup 18, such as MYB33 and MYB101, have also been shown to be redundant in the regulation of ABA signaling during germination (Reyes and Chua 2007).

It is particularly interesting to explore in the future the mechanism by which the MYB37 protein functions to regulate development of the flower organs and seed productivity. In regard to this aspect, it has been observed that, similar to the *MYB37*-overexpressing lines, *Arabidopsis* transgenic plants overexpressing several ABA-responsive genes such as *ABF4*, *DREB1A* and *MYB44* exhibited both enhanced drought tolerance and delayed flowering (Kasuga et al. 1999; Gilmour et al. 2000; Kang et al. 2002; Jung et al. 2008), which suggests an important role of ABA in the regulation of floral transition. Additionally, promotion of seed production by MYB37 overexpression seems to be associated with prolonged vegetative phase, which may allow producing bigger biomass by prolonged life of photosynthesis of leaves (Fig. 3). It is interesting to answer this open question in the future to explore its significance in crop production in agriculture.

## Electronic supplementary material

Supplementary material 1 (PDF 593 kb)
