# Supplementary material for: Overexpression of the MYB37 transcription factor enhances abscisic acid sensitivity, and improves both drought tolerance and seed productivity in Arabidopsis thaliana
Source: Plant Mol Biol. 2015 Dec 8;90:267–79. doi: 10.1007/s11103-015-0411-1 (PMC4717180; doi:10.1007/s11103-015-0411-1)
Supplement: Supplementary file 1 — Supplementary material 1 (PDF 593 kb) [file 11103_2015_411_MOESM1_ESM.pdf]

Supplementary data

Yu *et al.*, Overexpression of the MYB37 transcription factor enhances abscisic acid sensitivity, and improves both drought tolerance and seed productivity in *Arabidopsis thaliana*

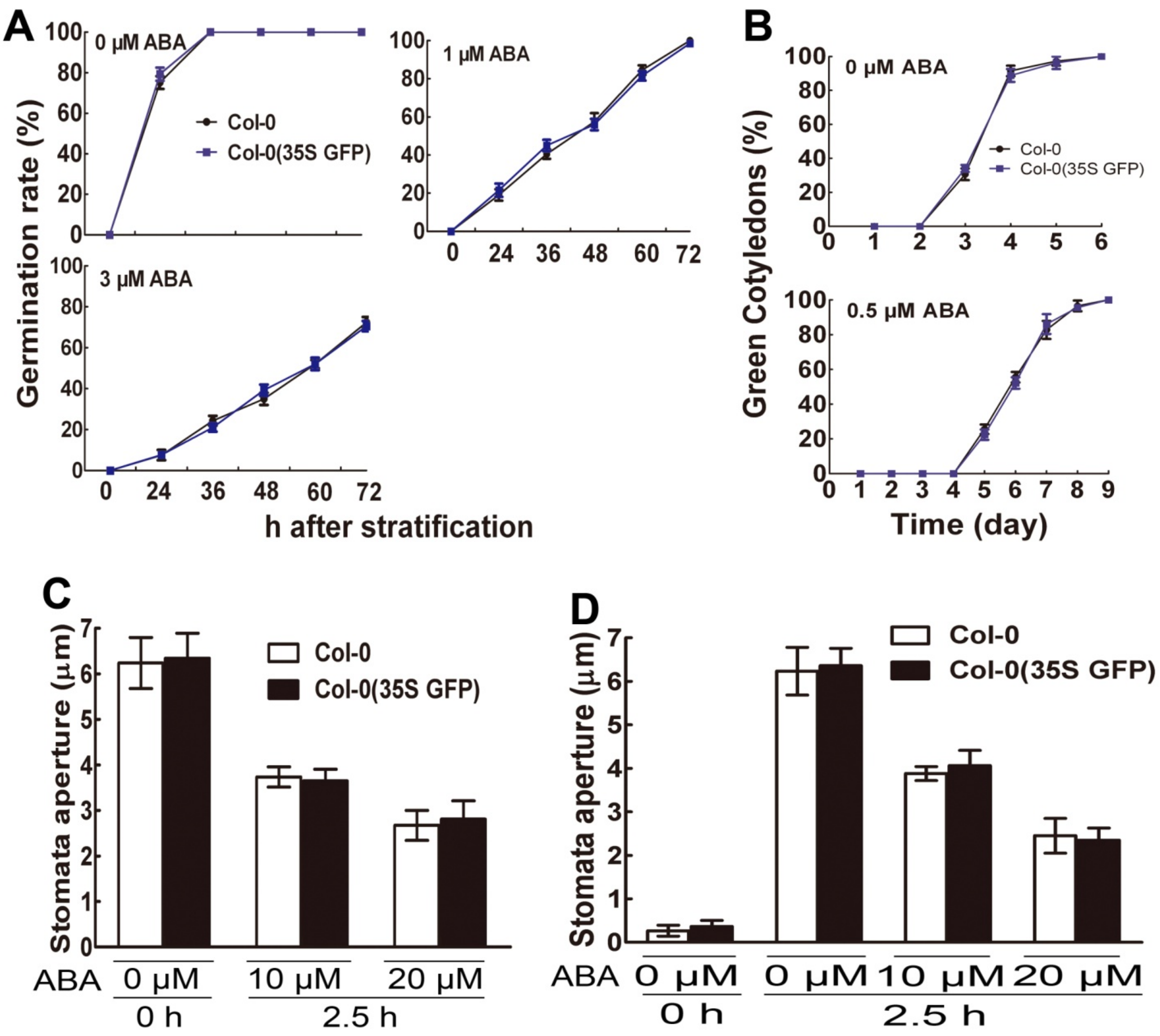

Supplementary Fig. S1. Transgenic plants expressing GFP show wild-type response to ABA. Statistics of the seed germination rates **(A)**, the rates of green cotyledons **(B)**, and ABA-induced stomatal closure **(C)** and inhibition of stomatal opening **(D)** of the wild-type Col-0 plants and the transgenic plants expressing GFP (Col-0 GFP). Each value is the mean ± SE of three biological determinations, and no significant difference was found at P<0.05 (Duncan's multiple-range test) when comparing the germination rates (A), cotyledons greening rates (B) and stomatal apertures in response to ABA treatment between the two genotypes.

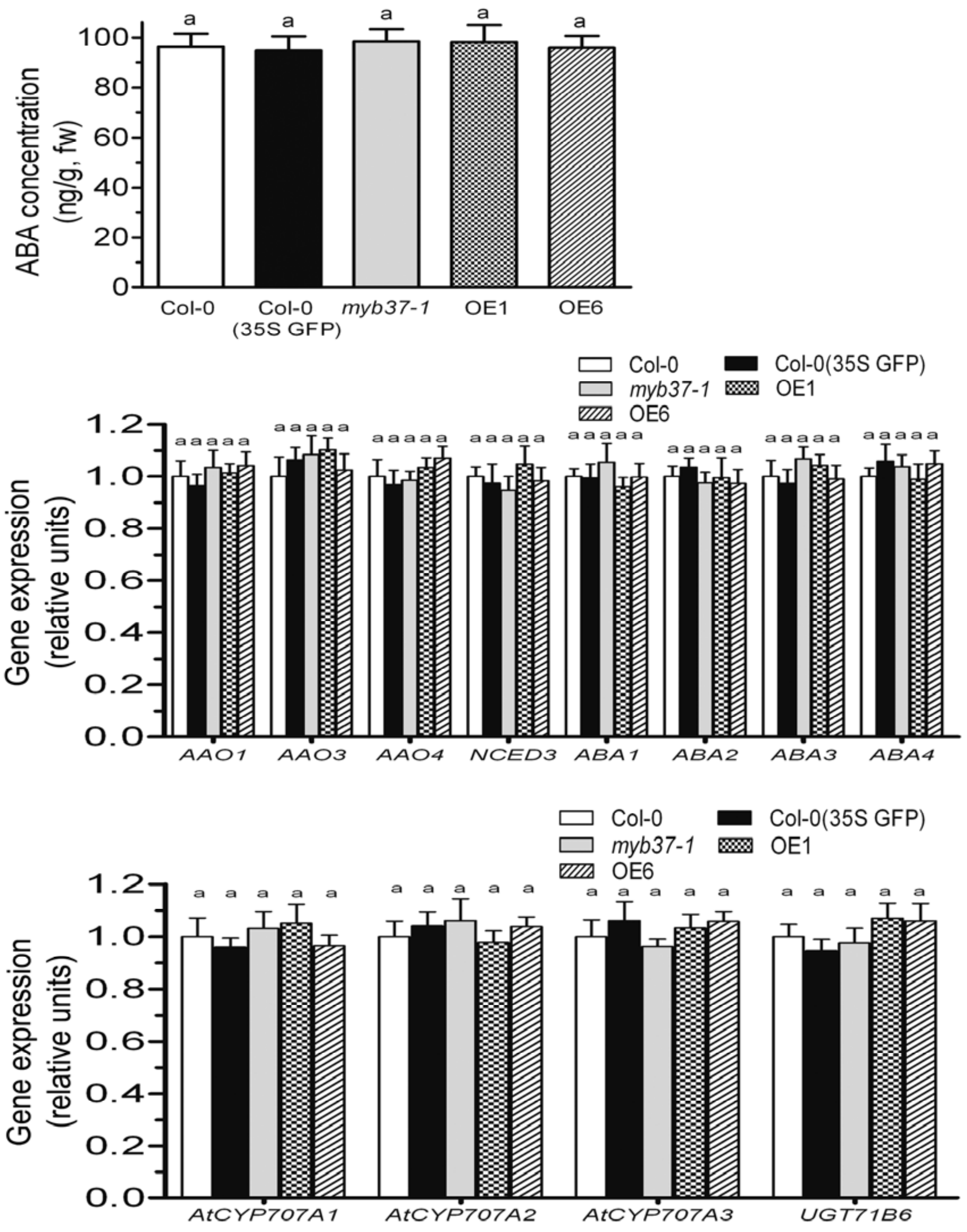

Supplementary Fig. S2. Changes in *MYB37* expression alter neither endogenous ABA concentration nor expression of ABA metabolism-related genes. **Top panel:** ABA concentrations in the 24 h-imbibed seeds of different genotypes (wild-type Col-0, control transgenic line expressing GFP only, *myb37-1* knockout mutant allele, and two *MYB37*-everexpression lines OE1 and OE6). Each value is the mean ± SE of three biological determinations, and the same letters indicate non-significant differences at $P<0.05$ (Duncan's multiple-range test) when comparing the ABA concentrations among different genotypes. ABA concentrations were assayed by ELISA method using a commercial immunoassay detection kit for ABA (Plant Growth Regulator Immunoassay Detection Kit, PGR1, Sigma) according to the procedures described previously (Mei C, Jiang SC, Lu YF, Wu FQ, Yu YT, Liang S, Feng XJ, Portoles Comeras S, Lu K, Wu Z, Wang XF, Zhang DP, 2014: *Arabidopsis* pentatricopeptide

repeat protein SOAR1 plays a critical role in abscisic acid signalling. J Exp Bot 65:5317-5330). **Middle and bottom panels:** Expression of ABA metabolism-related genes in different genotypes. RNA sample was extracted from the 24 h-imbibed seeds of different genotypes (wild-type Col-0, control transgenic line expressing GFP only, *myb37-1* knockout mutant allele, and two *MYB37*-everexpression lines OE1 and OE6) after three-day stratification, and the gene expression levels of ABA biosynthetic enzyme-encoding genes *AAO1*, *AAO3*, *AAO4*, *NCED3* and *ABA1-4*, as well as ABA catabolic enzyme-encoding genes *AtCYP707A1*, *AtCYP707A2*, *AtCYP707A3* and *UGT71B6* were analyzed by real-time PCR. The gene expression levels were relative units normalized relative to the value from the sample of the wild-type Col-0 plants (as 1). "Relative units" for the gene expression are normalized relative to the value of wild-type Col-0, which is taken as 1. Each value is the mean ±SE of three biological determinations, and the same letters indicate non-significant differences at $P<0.05$ (Duncan's multiple range test) when comparing the same gene among different genotypes.

Supplementary Table 1. Primers used for cloning cDNAs and screening mutants.

LP, left genomic primer; RP, right genomic primer; LBb1.3, left border primer of the T-DNA insertion

**Generation of transgenic plants (*MYB37*)**<br>forward primer:5’-CTAGTCTAGAATGGGAAGAGCTCCGTGTT-3’<br>reverse primer:5’-CGGGGTACCGGAGTAGAAATAGGGCAAGC-3’
**Screening of loss-of function mutant**<br>LP: 5’-TCGGACATTTCAGTTTGGAAG-3’<br>RP: 5’-TGTGAAAAGACCAACCTCACC-3’<br>LBb1.3: 5’-ATTTTGCCGATTTCGGAAC-3’

Supplementary Table 2. Gene-specific primers for real-time PCR analysis

Gene name	Primers	
ACTIN2/8<br>(a control)	forward primer:<br>reverse primer:	5'-GGTAACATTGTGCTCAGTGGTGG-3'<br>5'-AACGACCTTAATCTTCATGCTGC-3'
MYB37<br>(AT5G23000)	forward primer:<br>reverse primer:	5'-CGACAAGACAAAAGTGAAGCGA-3'<br>5'-TGGCAGCGAAGAGACTAAAAATG-3'
ABF1<br>(At1g49720)	forward primer:<br>reverse primer:	5'-TCAACAACTTAGGCGGCGATAC-3'<br>5'-GCAACCGAAGATGTAGTAGTCA-3'
ABF2<br>(At1g45249)	forward primer:<br>reverse primer:	5'-TTGGGGAATGAGCCACCAGGAG-3'<br>5'-GACCCAAAATCTTTCCCTACAC-3'
ABF3<br>(At4g34000)	forward primer:<br>reverse primer:	5'-CTTTGTTGATGGTGTGAGTGAG-3'<br>5'-GTGTTTCCACTATTACCATTGC-3'
ABF4<br>(At3g19290)	forward primer:<br>reverse primer:	5'-AACAACTTAGGAGGTGGTGGTC-3'<br>5'-CTTCAGGAGTTCATCCATGTTC-3'
ABI1<br>(At 4g26080)	forward primer:<br>reverse primer:	5'-AGAGTGTGCCTTTGTATGGTTTTA-3'<br>5'-CATCCTCTCTCTACAATAGTTCGCT-3'
ABI2<br>(At5g57050)	forward primer:<br>reverse primer:	5'-GATGGAAGATTCTGTCTCAACGATT-3'<br>5'-GTTTCTCCTTCACTATCTCCTCCG-3'
ABI4<br>(At2g40220)	forward primer:<br>reverse primer:	5'-GGGCAGGAACAAGGAGGAAGTG-3'<br>5'-ACGGCGGTGGATGAGTTATTGAT-3'
ABI5<br>(At2g36270)	forward primer:<br>reverse primer:	5'-CAATAAGAGAGGGATAGCGAACGAG-3'<br>5'-CGTCCATTGCTGTCTCCTCCA-3'
COR15A<br>(AT2G42540)	forward primer:<br>reverse primer:	5'-GTGACGGATAAAACAAAAGAGG-3'<br>5'-GACCCTACTTTGTGGCATCCTT-3'
COR15B<br>(AT2G42530)	forward primer:<br>reverse primer:	5'-AAAGCAGAGTGGTGTTGGTACCGT-3'<br>5'-TCATCGAGGATGTTGCCGTCACTT-3'
COR47<br>(AT1G20440)	forward primer:<br>reverse primer:	5'-ATGGCTGAGGAGTACAAGAACAACGTT-3'<br>5'-TCTTCTTCTTCTTCTCCTTCTTTTCCT-3'
DREB1A<br>(At4g25480)	forward primer:<br>reverse primer:	5'-GATCAGCCTGTCTCAATTTC-3'<br>5'-CTTCTGCCATATTAGCCAAC-3'
DREB2A<br>(At5g05410)	forward primer:<br>reverse primer:	5'-AAGGTAAAGGAGGACCAGAG-3'<br>5'-ACACAACCAGGAGTCTCAAC-3'
EM1<br>At3g51810)	forward primer:<br>reverse primer:	5'-CAAAGCAACTGAGCAGAGAAGAGC-3'<br>5'-CCTCCCTTGCTCCTTCCTTCA-3'
EM6<br>(At2g40170)	forward primer:<br>reverse primer:	5'-CAGCAGATGGGACGCAAAGG-3'<br>5'-TATTACATCCGTGTGGGGAAGTTTG-3'
ERD10<br>(At1g20450)	forward primer:<br>reverse primer:	5'-TCTCTGAACCAGAGTCGTTT-3'<br>5'-CTTCTTCTCACCGTCTTCAC-3'
KIN1<br>(At5g15960)	forward primer:<br>reverse primer	5'-ACCAACAAGAATGCCTTCCA-3'<br>5'-CCGCATCCGATACACTCTTT-3'
KIN2<br>(At5g15970)	forward primer:<br>reverse primer:	5'-ACCAACAAGAATGCCTTCCA-3'<br>5'-ACTGCCGCATCCGATATACT-3'
MYC2<br>(At1g32640)	forward primer:<br>reverse primer:	5'-TCATACGACGGTTGCCAGAA-3'<br>5'-AGCAACGTTTACAAGCTTTGATTG-3'

RAB18<br>(At5g66400)	forward primer:<br>reverse primer:	5’-CAGCAGCAGTATGACGAGTA-3’<br>5’-CAGTTCCAAAGCCTTCAGTC-3’
RD22<br>(AT5G25610)	forward primer:<br>reverse primer:	5’-ACTTGGTAAATATCACGTCAGGGCT-3’<br>5’-CTGAGGTGTTCTTGTGGCATACC-3’
RD26<br>(AT4G27410)	forward primer:<br>reverse primer:	5’-GAAGGTGAGGCGGAGAGTG-3’<br>5’-CCCGAAACTCTGAGTCAACCT-3’
RD29A<br>(At5g52310)	forward primer:<br>reverse primer:	5’-ATCACTTGGCTCCACTGTTGTTC-3’<br>5’-ACAAAACACACATAAACATCCAAAGT-3’
RD29B<br>(AT5G52300)	forward primer:<br>reverse primer:	5’-CTTGGCACCACCGTTGGGACTA-3’<br>5’-TCAGTTCCCA GAATCTTGAACT-3’
SnRK2.2<br>(At3g50500)	forward primer:<br>reverse primer:	5’-ATATGCCATCGGGATCTGAA-3’<br>5’-TTGGTTGGGAATGAAGAACAG-3’
SnRK2.3<br>(At5g66880)	forward primer:<br>reverse primer:	5’-GTTGGATGGAAGTCCTGCTC-3’<br>5’-TGCCATCATATTCCTGACGA-3’
SnRK2.6<br>(At4g33950)	forward primer:<br>reverse primer:	5’-TGGAGTTGCGAGATTGATGAGAG-3’<br>5’-CCTGTGGTTGATTATCTCCCTTTTT-3’
AAO1<br>(AT5G20960)	forward primer:<br>reverse primer:	5’-TGCTCTGTTTTTGTTTGTGGTTTG-3’<br>5’-CACAATCCATTGCCATCGACAT-3’
AAO3<br>(AT2G27150)	forward primer:<br>reverse primer:	5’-TCCATCATGGACTGCTCCTTC-3’<br>5’-CGAGACACTAGCGCCAAGAAA-3’
AAO4<br>(AT1G04580)	forward primer:<br>reverse primer:	5’-CAACAGTGGTCATCACAAGAACC-3’<br>5’-GGAACCGGTAACTCAAAACCAAG-3’
NCED3<br>(AT3G14440)	forward primer:<br>reverse primer:	5’-AGCCGCCATTATCGTCTTCTC-3’<br>5’-GGAGTGTGAAGCGCAGATGAA-3’
ABA1<br>(AT5G67030)	forward primer:<br>reverse primer:	5’-TCACTGACAAAGCCGATGACC-3’<br>5’-CGAAACGCAACAATCGTCG-3’
ABA2<br>(AT1G52340)	forward primer:<br>reverse primer:	5’-AAGCATGAAACATGCAGCTCG-3’<br>5’-AAGAATGTGGACCAACGCCTC-3’
ABA3<br>(AT1G16540)	forward primer:<br>reverse primer:	5’-TAGTGAAGAAATCCAATGAGCCCT-3’<br>5’-CTTCTTCCCCAACTCCAATCCAA-3’
ABA4<br>(AT1G67080)	forward primer:<br>reverse primer:	5’-GACTCTTGCTTCTGCTTGGATTC-3’<br>5’-AGAAGGCAAAGTGAAACCGAATG-3’
AtCYP707A1<br>(At4g19230)	forward primer:<br>reverse primer:	5’-CAAAACCCAATACGTTCATGCCA-3’<br>5’-CTCGCTCCAACAATTGACCAAAC-3’
AtCYP707A2<br>(At2g29090)	forward primer:<br>reverse primer:	5’-CAAGAAAAATGCCACTGACCACT-3’<br>5’-TCCAACCCTTTGGGATCAAGTAG-3’
AtCYP707A3<br>(At5g45340)	forward primer:<br>reverse primer:	5’-TCTCAATACTCGGCAAAGACGAA-3’<br>5’-GAGCTTTCATGGCTTTGTGGAAT-3’
UGT71B6<br>(AT3G21780)	forward primer:<br>reverse primer:	5’-GGTTCAGTGAGGAACAAGTGAGA-3’<br>5’-TAACCTTTCCTCTGTTAGCCGTC-3’
